# 611. Increased isavuconazole loading dosage yields earlier attainment of serum target concentrations in critical ill patients with extracorporeal membrane oxygenation

**DOI:** 10.1093/ofid/ofad500.677

**Published:** 2023-11-27

**Authors:** Stefan Hatzl, Lisa Kriegl, Florian Posch, Andreas Meinitzer, Gernot Schilcher, Philipp Eller, Yvonne Grinschgl, Tina Muhr, Martin Hoenigl, Robert Krause

**Affiliations:** Intensive Care Unit, Department of Internal Medicine, Medical University of Graz, Graz, Austria, Graz, Steiermark, Austria; Division of Infectious Diseases, Department of Internal Medicine, Medical University of Graz, Graz, Austria, Graz, Steiermark, Austria; Division of Hematology, Department of Internal Medicine, Medical University of Graz, Graz, Austria, Graz, Steiermark, Austria; Clinical Institute of Medical and Chemical Laboratory Diagnostics, Medical University of Graz, Graz, Austria, Graz, Steiermark, Austria; Intensive Care Unit, Department of Internal Medicine, Medical University of Graz, Graz, Austria, Graz, Steiermark, Austria; Intensive Care Unit, Department of Internal Medicine, Medical University of Graz, Graz, Austria, Graz, Steiermark, Austria; Department of Anesthesiology and Intensive Care Medicine, Medical University of Graz, Graz, Austria, Graz, Steiermark, Austria; Department of Internal Medicine, Landeskrankenhaus Graz 2, Graz, Austria, Graz, Steiermark, Austria; Division of Infectious Diseases, Department of Internal Medicine, Medical University of Graz, Graz, Austria, Graz, Steiermark, Austria; Medical University of Graz, Section of Infectious Diseases and Tropical Medicine, Department of Internal Medicine, Graz, Steiermark, Austria

## Abstract

**Background:**

Invasive fungal infections (IFI) are a major threat for critical ill patients admitted to the intensive care unit (ICU). Isavuconazole is a triazole antifungal agent recommended for treatment of invasive aspergillosis or mucormycosis. Both IFIs might complicate influenza or COVID-19 in ICU patients. Treatment of acute respiratory failure in those patients may require extracorporeal membrane oxygenation (ECMO). The appropriate dosage of selected antifungal drugs in ECMO patients is of utmost importance for successful antifungal prophylaxis or treatment of IFIs. Isavuconazole plasma concentrations < 1 µg/mL were reported in ECMO patients up to 24h after the first 200mg loading dose.

**Methods:**

Critically ill ECMO patients receiving isavuconazole (first loading dose either 200mg, 300mg or 400mg, followed by isacuconazole administration as recommended) were included. Isavuconazole plasma concentrations were measured starting 2 h after the first isavuconazole loading dose up to 168 h.

**Results:**

Out of 15 ECMO patients in total (median 62 years, range 47-64; 7 (47%) female) seven (46%) received standard isavuconazole loading regimen with 200mg as first dose, 3 (20%) received 300mg isavuconazole as first loading dose and 5 (34%) received 400mg isavuconazole as first loading dose. Twelve patients (80%) had veno-venous ECMO, 2 veno-arterial and one veno-arterial-venosus ECMO cannulation. The patients had a median p_a_O2/FiO_2_ ratio of 76 (range 68-87) and sequential organ failure assessment score of 8.5. Three patients received isavuconazol for antifungal prophylaxis and 12 for treatment of IFI (Table 1). In comparison to patients receiving a first loading dose of 200mg or 300mg the median plasma concentrations in patients receiving the first loading dose of 400mg were significantly higher and constantly above 1µg/ml up to the first 24h. (Figure 1). No adverse effects related to isavuconazole were observed. No patient had isavuconazole concentrations ≥ 5µg/mL.

Isavuconazole plasma concentrations in ECMO patients

**Figure:**
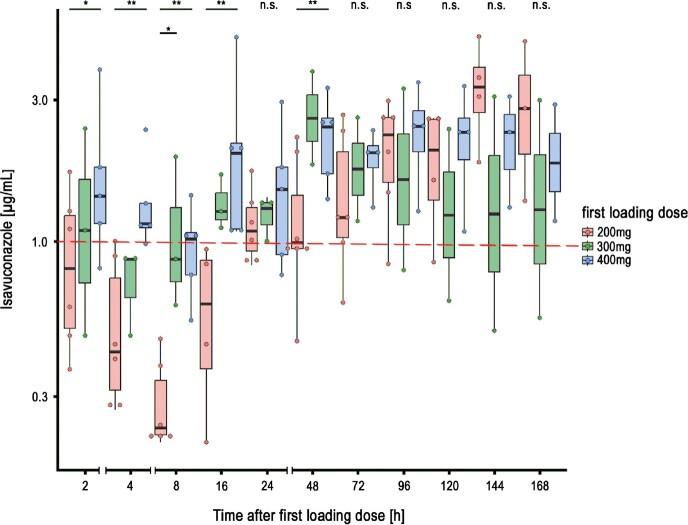

Isavuconazole plasma concentrations in ECMO patients at given timepoints after first isavuconazole dose. At dedicated isavuconazole administration timepoints, samples were obtained just before the next scheduled isavuconazole infusions.

Clinical and laboratory characteristics of the study population of 15 patients receiving isavuconazole and ECMO

**Figure:**
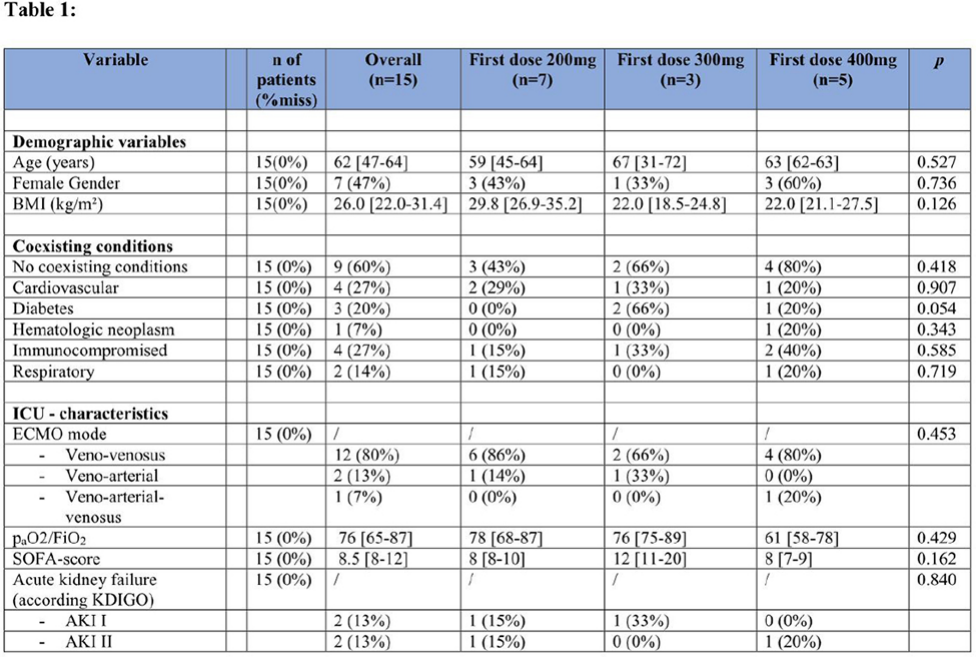

**Conclusion:**

Increasing the first loading dose of isavuconazole from 200 to 400mg led to a substantial increase in median isavuconazole plasma concentrations achieving ≥ 1µg/mL constantly after the first loading dose and was not associated with toxic side effects.

**Disclosures:**

**Robert Krause, MD**, Pfizer: Grant/Research Support

